# Non-Secreted Mature Decoy-Resistant IL-18-Armed Oncolytic Vaccinia Virus Elicits Potent Antitumor Effects in an Aggressive Murine Ovarian Cancer Model

**DOI:** 10.3390/cancers18071065

**Published:** 2026-03-25

**Authors:** Pingpo Ming, Chunyan Li, Junjie Ye, Lingjuan Chen, Julia Waltermire, Jinshun Zhao, Maya Eid, Ting Zhang, Wei Ge, Jinghua Ren, David L. Bartlett, Zuqiang Liu

**Affiliations:** 1 Allegheny Health Network Cancer Institute, Pittsburgh, PA 15212, USA; 2 Taikang Tongji (Wuhan) Hospital, Wuhan 430060, China; 3 Department of Obstetrics, Renmin Hospital of Wuhan University, Wuhan 430060, China; 4 Department of Surgery, Drexel University College of Medicine, Philadelphia, PA 19104, USA; 5 Department of Cancer Center, Renmin Hospital of Wuhan University, Wuhan 430060, China; 6 Cancer Center, Union Hospital, Huazhong University of Science and Technology, Wuhan 430023, China; 7 College of Arts & Science, Vanderbilt University, Nashville, TN 37235, USA

**Keywords:** ovarian cancer, oncolytic virus, vaccinia virus, nsmDR-18, spleen, peritoneal lavage-derived cells, tumor microenvironment, PD-1

## Abstract

**Background/Objectives**: Ovarian cancer is the most lethal gynecologic malignancy, largely due to late diagnosis and the high prevalence of malignant ascites, a hallmark of advanced disease that is difficult to control and contributes to immune suppression and treatment failure. Despite advances in standard care, durable responses are rare. This study investigates a novel immunotherapeutic strategy designed to overcome the suppressed peritoneal microenvironment using an oncolytic vaccinia virus engineered to express a decoy-resistant IL-18 mutein. **Methods**: We generated a vaccinia virus (vvDD-nsmDR-18) expressing a non-secreted, mature, decoy-resistant IL-18. Viral expression was validated via RT-qPCR and fluorescence microscopy, while cytotoxicity was confirmed using CCK-8 assays. The antitumor efficacy of vvDD-nsmDR-18 was evaluated in the aggressive murine ID8a ovarian cancer model. The underlying mechanisms of action were investigated using flow cytometry and transcriptional profiling. **Results**: Treatment with vvDD-nsmDR-18 significantly prolonged survival and was associated with reduced abdominal distension consistent with decreased ascites burden. Immune analyses indicated enhanced T cell activation across multiple anatomical compartments, including tumors, peritoneal cavity, and spleens, the latter recently suggested to serve as a reservoir for tumor-reactive T cells. This systemic activation was characterized by increased IFN-γ and perforin expression. In addition, vvDD-nsmDR-18 treatment was associated with expansion of CD39^+^CD103^+^CD8^+^ tumor-reactive T cells and a shift toward a lower PD-1 expression phenotype within this population. **Conclusions**: These findings demonstrate that nsmDR-18-expressing oncolytic viruses can remodel the immunosuppressive landscape of advanced ovarian cancer, suggesting this approach is a promising candidate for further clinical development.

## Simple Summary

Ovarian cancer is often diagnosed at advanced stages and frequently leads to the buildup of fluid in the abdomen, called ascites, which causes discomfort and weakens the body’s ability to fight the tumor. New treatment strategies are needed to improve outcomes for patients with advanced disease. In this study, we tested a modified virus designed to selectively infect tumors and stimulate the immune system. The virus was engineered to produce a form of the immune-activating protein interleukin-18 that helps boost immune responses. In a mouse model of aggressive ovarian cancer, treatment with this engineered virus improved survival and reduced abdominal swelling associated with fluid accumulation. The therapy was also linked to increased activation of immune cells that can recognize and attack tumors. These findings suggest that this virus-based approach may help strengthen antitumor immunity and could be further developed as a potential treatment strategy for ovarian cancer.

## 1. Introduction

Ovarian cancer remains the most lethal gynecologic malignancy in the United States, with an estimated 21,010 new cases and 12,450 deaths projected in 2026 [1]. Globally, approximately 324,398 new cases and 206,839 deaths were reported in 2022 [2]. Ovarian cancers arise from the uncontrolled growth of malignant cells originating in the ovaries, fallopian tubes, or peritoneum. Early detection is challenging, as symptoms are often nonspecific or absent until the disease has progressed to advanced stages.

When diagnosed at an early, localized stage, ovarian cancer is highly treatable, with a 5-year survival rate of approximately 92% based on data from 2015–2021. However, nearly 75% of patients present with advanced-stage disease at the time of diagnosis, resulting in a marked decline in survival outcomes. For patients with regional disease involving adjacent organs, the 5-year survival rate decreases to approximately 71%, while those with distant metastatic disease have a 5-year survival rate of only 32% during the same period [1].

Despite advances in cytoreductive surgery and platinum-based chemotherapy, durable disease control for advanced ovarian cancer remains elusive, with high rates of recurrence and therapy resistance. These limitations highlight the urgent need for novel therapeutic approaches capable of overcoming immune suppression and heterogeneity within the peritoneal tumor microenvironment. In this context, cancer immunotherapy, and especially oncolytic virus-based approaches, holds considerable promise by integrating selective oncolysis with localized immune activation in the peritoneal cavity, offering a rational strategy to address peritoneal carcinomatosis and malignant ascites in advanced ovarian cancer.

Oncolytic viruses (OVs) are replication-competent viruses that selectively infect and kill cancer cells while sparing normal, healthy cells. OVs, especially those that are appropriately armed with immunomodulatory molecules, have the potential to convert “cold” tumors into “hot” ones, thereby eliciting potent antitumor effects alone or in combination with other anticancer agents [3,4,5,6,7,8,9,10,11,12,13]. A wide range of chemokines, cytokines, or other immune regulatory molecules have been incorporated into OVs to reshape the tumor microenvironment and enhance antitumor effects [14,15,16,17,18,19,20,21,22,23,24,25,26,27,28,29,30,31,32,33,34,35,36,37,38,39,40,41,42,43,44,45].

Among these candidates, interleukin-18 (IL-18) has emerged as a particularly promising cytokine for cancer immunotherapy. IL-18 plays a pivotal role in immune responses, particularly in enhancing the activity of natural killer (NK) cells and T cells, as well as in promoting the production of IFN-γ [46]. IL-18 signaling requires a heterodimeric receptor composed of the inducible IL-18Rα chain and the constitutively expressed IL-18Rβ co-receptor. However, IL-18 activity is tightly regulated by a high-affinity soluble decoy receptor, IL-18 binding protein (IL-18BP), which sequesters IL-18 and prevents receptor engagement, thereby limiting its therapeutic efficacy [47].

To overcome this limitation, a decoy-resistant IL-18 (DR-18) variant that retains full signaling capacity while evading IL-18BP inhibition was engineered [48]. DR-18 displayed on the surface of non-pathogenic *Escherichia coli* elicited potent antitumor effects in murine models of colon cancer and melanoma [49]. Similarly, intratumoral delivery of DR-18 via an oncolytic adenovirus induced robust antitumor responses across multiple subcutaneous tumor models, which were further enhanced by PD-1 blockade [50]. More recently, building on the clinically validated vvDD oncolytic vaccinia virus (oVV) platform [51], we developed an extremely potent non-secreted mutant of DR-18 (nsmDR-18) and demonstrated that intratumoral delivery of nsmDR-18 via an oVV (vvDD-nsmDR-18) resulted in potent antitumor activity with reduced systemic toxicity [52].

In the present study, we investigated whether and how vvDD-nsmDR-18 elicits effective antitumor immune responses in an aggressive murine ovarian cancer model, with a particular focus on its impact within the peritoneal tumor microenvironment.

## 2. Material and Methods

### 2.1. Cell Lines

Cell lines used in this study included normal African green monkey kidney fibroblast CV1 cells (#CCL-70™) and human embryonic kidney 293 (HEK293) cells (#CRL-1573™), both acquired from the American Type Culture Collection (ATCC) (Manassas, VA, USA). The aggressive murine ovarian cancer cell line ID8a-luc was generated in our laboratory [15], and the human ovarian cancer cell line A2780 was a kind gift from the National Cancer Institute, USA. For virus preparation, HEK293 cells were cultured in Dulbecco’s Modified Eagle’s Medium (DMEM; Thermo Fisher Scientific, Waltham, MA, USA, #11965092) supplemented with 15% newborn calf serum (Sigma, St. Louis, MO, USA, #N4637), 2 mM L-glutamine (Thermo Fisher Scientific, Waltham, MA, USA, #A2916801), and 1× penicillin/streptomycin (Thermo Fisher Scientific, Waltham, MA, USA, #10378016) at 37 °C with 5% CO_2_. All other cell lines were grown in DMEM containing 10% fetal bovine serum (FBS; Sigma, St. Louis, MO, USA, #F0926), 2 mM L-glutamine (Thermo Fisher Scientific, Waltham, MA, USA, #A2916801), and 1× penicillin/streptomycin (Thermo Fisher Scientific, Waltham, MA, USA, #10378016), maintained in the same incubator conditions.

### 2.2. Virus Replication and nsmDR-18 Expression

An oncolytic vaccinia virus vvDD-nsmDR-18 which is expressing non-secreted mature decoy-resistant IL-18 mutein was generated in our lab as previously described [52]. The cDNAs encoding nsmDR-18 and marker yellow fluorescent protein (YFP) were inserted at viral *thymidine kinase* (*tk*) locus. A double viral gene-inactivated (*tk-* and *vgf-*) oVV carrying *yfp* cDNA at its *tk* locus, vvDD-YFP (hereafter referred to as vvDD), was used as the control virus in this study. Viruses were propagated in HEK293 cells, purified by sucrose gradient centrifuge, and titrated in CV cells using standard plaque assay. ID8a-luc (2.0 × 10^5^/well), or A2780 (3.0 × 10^5^/well) cells were seeded in 24-well culture plates on day 0. On day 1, the seeded cells were treated with PBS or infected with vvDD or vvDD- nsmDR-18 at an MOI of 1 in 0.15 mL of 2% FBS-containing-DMEM per well for 2 h, after which 0.35 mL of 10% FBS-containing DMEM was added into each well. The cells were cultured for 24 h post-treatment and then incubated with Hoechst 33342 dye (Biotium, Fremont, CA, USA, #40046; 1 ug/mL) for 5–15 min before imaging. Virus replication and YFP expression were confirmed via fluorescence microscopy. Subsequently, RNA was extracted from harvested cells to quantify nsmDR-18 and viral gene A34R expression using RT-qPCR. Five–six-week-old female C57BL/6 (B6) mice were obtained from The Jackson Laboratory (Bar Harbor, ME) and maintained in a specific pathogen-free environment at the Allegheny Health Network Research Institute Preclinical Facility. All animal procedures received approval from the Allegheny Health Network Research Institute Institutional Animal Care and Use Committee. B6 mice were intraperitoneally (i.p.) inoculated with 3.5 × 10^6^ ID8a-luc cancer cells. Tumor-bearing mice were monitored by in vivo bioluminescence imaging (IVIS) using a Xenogen IVIS 200 Optical In Vivo Imaging System (Caliper Life Sciences, Hopkinton, MA, USA). On day 5 after tumor cell inoculation, mice were imaged and allocated to three treatment groups to achieve comparable baseline bioluminescence signal intensity. Each group received an i.p. injection of either vvDD, vvDD-nsmDR-18, or Phosphate-Buffered Saline (PBS). Tumors were harvested on day 5 after treatment for subsequent RT-qPCR analysis.

### 2.3. Cytotoxicity Assay In Vitro

ID8a-luc (5.0 × 10^3^/well), or A2780 (1.0 × 10^4^/well) cells were seeded in 96-well culture plates and treated with PBS or infected with indicated viruses the following day at different MOIs. The cells were cultured for 48 h posttreatment and then incubated with Hoechst 33342 dye (1 ug/mL) for 5–15 min before imaging. Cell viability was validated under a fluorescence microscope and determined using the Cell Counting Kit-8 (CCK-8) assay (Boster Biological Technology, Pleasanton, CA, USA).

### 2.4. Rodent Tumor Models

B6 mice were inoculated i.p. with 3.5 × 10^6^ ID8a-luc cancer cells. Tumor-bearing mice were monitored by IVIS. On day 5 after tumor cell inoculation, mice were imaged and allocated to treatment groups to achieve comparable baseline bioluminescence signal intensity, thereby minimizing potential differences in initial tumor burden. All animals meeting the inclusion criteria were included in the analysis and no animals were excluded based on experimental outcomes. Mice were then injected i.p. with vvDD, vvDD-nsmDR-18, or PBS as indicated. Mouse body girths were measured in the indicated time points to monitor abdominal distension. In some experiments, mice were euthanized at defined time points for tissue collection and downstream analysis. For peritoneal lavage, each mouse was injected i.p. with 3 mL sterile PBS containing 2% FBS and the mouse abdomen was gently massaged for at least 30 s. The lavage fluid (PBS with peritoneal cells) was withdrawn for further analysis. Mouse spleens were collected, photographed, and mechanically dissociated to generate single-cell suspensions for subsequent analyses. Mice were monitored throughout the study for general clinical condition, including activity, posture, grooming behavior, and body condition. In this intraperitoneal ovarian cancer model, body weight was not used as an indicator of systemic toxicity, as weight changes are strongly confounded by ascites accumulation. Previous studies have demonstrated that mice with ascites may exhibit increased body weight independent of disease severity or distress, limiting its interpretability as a toxicity readout [53,54]. At necropsy, major organs, including liver, lungs, kidneys, and heart, were examined for gross pathological abnormalities.

### 2.5. Flow Cytometry

To prepare single-cell suspensions, approximately 200 mg of collected tumor tissue was incubated in RPMI 1640 medium (2% FBS, 1 mg/mL collagenase IV, 0.1 mg/mL hyaluronidase, 200 U/mL DNase I) at 37 °C for 1–2 h. Single-cell suspensions, derived from tumor tissues, spleen, or peritoneal lavage samples (when available from the same animals), were initially blocked with anti-CD16/32 antibody (clone 93, eBioscience, San Diego, CA, USA; #14-0161-85; 1:200). Surface markers were then stained using fluorochrome-conjugated antibodies: CD45 (BUV395, clone 30-F11, Thermo Fisher Scientific, Waltham, MA, USA; #36-304-518; 1:300), CD4 (BUV661, clone GK1.5, Thermo Fisher Scientific, Waltham, MA, USA; #37-600-4180; 1:300), CD8 (BV650, clone 53-6.7, BioLegend, San Diego, CA, USA; #100742; 1:300), PD-1 (BV605, clone 29F.1A12, BioLegend, San Diego, CA, USA; #135220; 1:300), CD39 (BUV805, clone 24DMS1, Thermo Fisher Scientific, Waltham, MA, USA; #36-803-9180; 1:300), CD103 (PerCP-eFluor 710, clone 2E7, Thermo Fisher Scientific, Waltham, MA, USA; #50-113-0773; 1:300), and IL-18Rα (CD218a) (Allophycocyanin (APC), clone A17071D, BioLegend, San Diego, CA, USA; #157904; 1:300). Following surface staining, intracellular IFN-γ was stained using an FITC-conjugated antibody (clone REA638, Miltenyi Biotec, Auburn, CA, USA; #130-117-668; 1:100). The Cyto-Fast™ Fix/Perm Buffer Set (BioLegend, San Diego, CA, USA; #426803) was used for intracellular staining. To distinguish PD-1^low^ and PD-1^high^ populations within the CD39^+^CD103^+^CD8^+^ T cell subset, PD-1 gating boundaries were established using fluorescence-minus-one (FMO) controls together with the distribution of PD-1 expression within the gated population. Cell viability was assessed with Zombie Aqua™ Fixable Viability Kit (BioLegend, San Diego, CA, USA; #423102). Samples were acquired on a NovoCyte Penteon Flow Cytometer System (Agilent, Santa Clara, CA, USA) and analyzed with FlowJo software (Version 10.8.1; Tree Star Inc., Ashland, Oregon, USA).

### 2.6. RT-qPCR

Total RNA was isolated from virus-infected cells, peritoneal lavage-derived cells, and tumor tissues using the RNeasy Kit (Qiagen, Valencia, CA, USA). One microgram of isolated RNA was reverse transcribed into cDNA. Subsequently, 25 to 50 ng of cDNA was used for TaqMan mRNA expression analysis on a Quantagene q225 qPCR System (Kubo Technology, Beijing, China). All TaqMan primers were sourced from Thermo Fisher Scientific (Waltham, MA, USA). Gene expression was normalized to the housekeeping gene HPRT1 and quantified as fold increase (2^−ΔCT^), where ΔCT represents CT_(Target gene)_ − CT_(HPRT1)_.

### 2.7. Statistics

Animal survival is shown using Kaplan–Meier survival curves and was statistically analyzed using a log-rank (Mantel–Cox) test (GraphPad Prism version 10.2). Other statistical analyses were performed using one-way ANOVA with Tukey’s multiple comparison test (GraphPad Prism version 10.2). Data are presented as means ± SD. In the figures, standard symbols are used: *p* < 0.05 (*); *p* < 0.01 (**); *p* < 0.001 (***), and *p* < 0.0001 (****); ns indicates not significant.

## 3. Results

### 3.1. nsmDR-18 Expression via oVV Does Not Impair Viral Replication In Vitro and In Vivo

**Figure 1:**
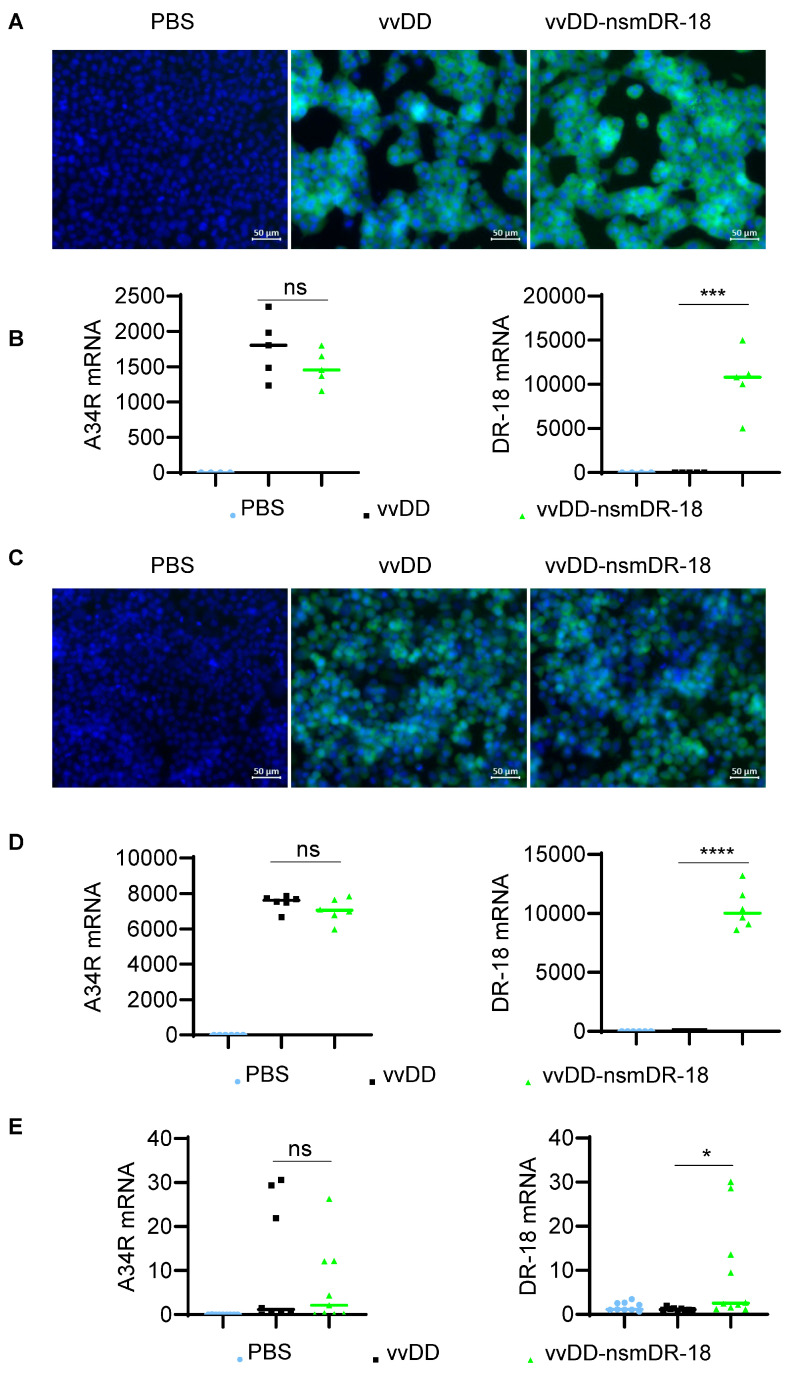
nsmDR-18 expression via oVV does not impair viral replication in vitro and in vivo. Tumor cells (ID8a-luc, 2.0 × 10^5^ cells; A2780, 3.0 × 10^5^ cells), were treated with PBS or infected with vvDD or vvDD-nsmDR-18 at an MOI of 1. Cells were stained with Hoechst 33342 and imaged. Representative fluorescence images of cells infected with YFP-expressing virus (yellow) and counterstained with Hoechst 33342 (blue) to visualize nuclei (**A**,**C**). Cells were harvested to measure A34R or nsmDR-18 expression using RT-qPCR 24 h posttreatment (**B**,**D**). B6 mice were inoculated i.p. with 3.5 × 10^6^ ID8a-luc cells and treated i.p. with the indicated viruses (1.0 × 10^8^ PFU/mouse) or PBS five days after tumor cell inoculation (4–6 mice/group). Tumors were harvested for RT-qPCR analysis. Expression of viral gene A34R and nsmDR-18 is shown in (**E**). Data show one representative experiment, except for panels in (**E**), which present combined data from two independent experiments. *p* < 0.05 (*); *p* < 0.001 (***), and *p* < 0.0001 (****); ns indicates not significant.

The infectivity and replication capacity of oVV were first assessed using the murine ovarian cancer cell line ID8a-luc. Cells were infected with vvDD-nsmDR-18 or control virus vvDD at an MOI of 1 or treated with PBS. Twenty-four hours posttreatment, cells were stained with Hoechst 33342 and imaged. Comparable YFP expression was observed in cells infected with either virus, whereas no signal was detected in PBS-treated cells (Figure 1A), indicating similar infectivity and replication between the two viruses in ID8a-luc cells.

To further quantify viral replication, total RNA was isolated from treated ID8a-luc cells and analyzed by RT-qPCR. Expression of the viral gene *A34R* did not differ significantly between vvDD-nsmDR-18- and vvDD-infected cells, whereas nsmDR-18 mRNA was detected exclusively and at significantly elevated levels in cells infected with vvDD-nsmDR-18 (Figure 1B). These data indicate that robust nsmDR-18 expression does not compromise viral replication. A similar expression pattern was observed in the human ovarian cancer cell line A2780 (Figure 1C,D).

To determine whether nsmDR-18 expression affects viral replication in vivo, we next evaluated viral gene and nsmDR-18 expression in ovarian tumors following treatment. B6 mice were injected i.p. with ID8a-luc tumor cells, and five days later, treated i.p. with PBS, vvDD, or vvDD-nsmDR-18. Tumor nodules were harvested on day 5 posttreatment, and total RNA was extracted for RT-qPCR analysis. Expression of the viral gene *A34R* was comparable between the two viral treatment groups, indicating similar levels of viral replication in vivo. In contrast, nsmDR-18 mRNA was significantly elevated only in tumors from vvDD-nsmDR-18–treated mice (Figure 1E). Collectively, these results demonstrate that nsmDR-18 expression does not impair oVV replication in vitro or in vivo.

### 3.2. nsmDR-18 Expression via oVV Does Not Impair Viral Cytotoxicity

**Figure 2:**
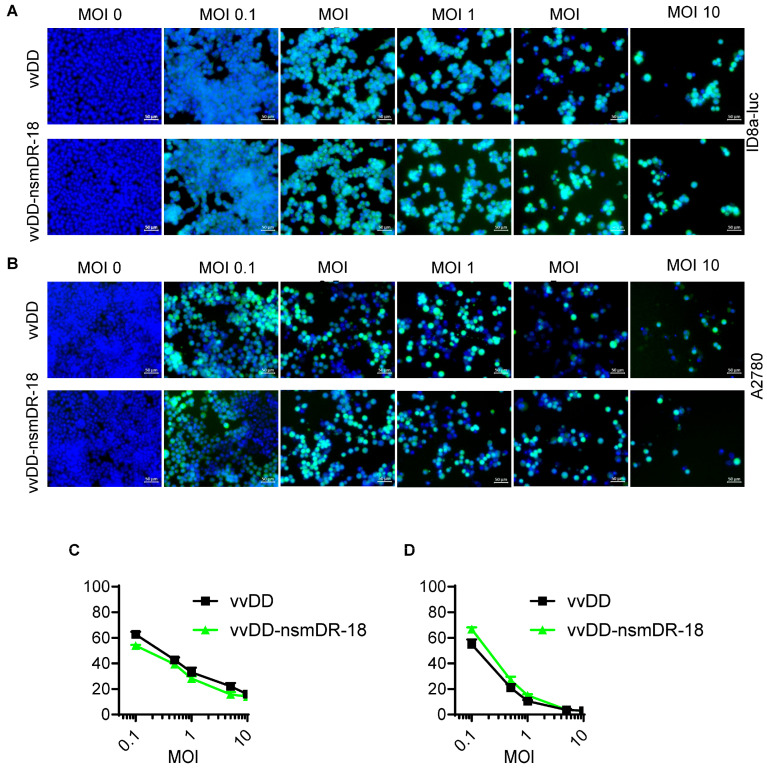
nsmDR-18 expression via oVV does not impair viral cytotoxicity. Tumor cells (ID8a-luc, 5.0 × 10^3^ cells; A2780, 1.0 × 10^4^ cells), were treated with PBS or infected with vvDD or vvDD-nsmDR-18 at indicated MOIs. Cells were stained with Hoechst 33342 and imaged. Representative fluorescence images of cells infected with YFP-expressing virus (yellow) and counterstained with Hoechst 33342 (blue) to visualize nuclei (**A**,**B**). Cell viability was measured 48 h post-infection (**C**,**D**). Data show one representative experiment.

To evaluate whether nsmDR-18 expression affects the direct cytolytic activity of oVV, ID8a-luc and A2780 cells were infected with vvDD-nsmDR-18 or control vvDD at MOIs of 0, 0.1, 0.5, 1, 5, or 10. Cell viability was assessed 48 h post-infection. Cells were stained with Hoechst 33342 and imaged to quantify cell numbers. In both cell lines, cell numbers decreased progressively with increasing viral dose, with no discernible difference between vvDD-nsmDR-18 and vvDD treatment (Figure 2A,B).

Cell viability was further quantified using the CCK-8 assay. Consistent with imaging results, both viruses induced comparable, dose-dependent reductions in cell viability in ID8a-luc and A2780 cells (Figure 2C,D). Together, these data indicate that nsmDR-18 expression does not alter the intrinsic oncolytic cytotoxicity of vvDD in ovarian cancer cells.

### 3.3. vvDD-nsmDR-18 Elicits Potent Antitumor Effects in a Murine Ovarian Cancer Model

We evaluated the in vivo antitumor efficacy of vvDD-nsmDR-18 in an aggressive murine ovarian cancer ID8a-luc model. B6 mice were injected i.p. with ID8a-luc tumor cells, and five days later, treated i.p. with PBS or 1.0 × 10^8^ of PFU of vvDD or vvDD-nsmDR-18. Tumor progression was monitored longitudinally using in vivo imaging. Survival was monitored until spontaneous death or euthanasia at predefined humane endpoints.

**Figure 3:**
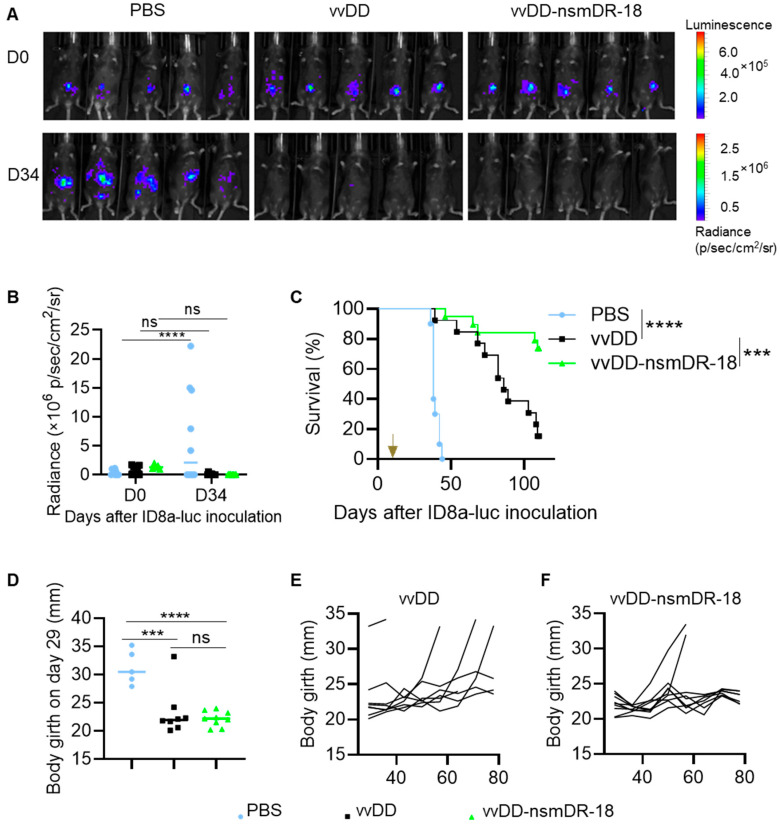
vvDD-nsmDR-18 elicits potent antitumor effects in an aggressive ovarian cancer model. B6 mice were inoculated i.p. with 3.5 × 10^6^ ID8a-luc cells and treated i.p. with the indicated viruses (1.0 × 10^8^ PFU/mouse) or PBS five days after tumor cell inoculation (the arrow indicates treatment initiation; 5–10 mice/group). Live animal imaging of representative mice with ID8a-luc tumors on day 34 after tumor cell inoculation (**A**), quantified data derived from the imaging (**B**) and Kaplan–Meier survival curves (**C**) are shown. The mouse body girth measured on day 29 after tumor cell inoculation for all treatment groups is shown in (**D**). Longitudinal changes in individual body girth from days 29–78 are shown for vvDD-treated mice in (**E**) and for vvDD-nsmDR-18-treated mice in (**F**). Data show one representative experiment, except for panel (**C**), which presents combined data from two independent experiments (*n* = 10 for PBS; *n* = 13 for vvDD; *n* = 19 for vvDD-nsmDR-18). *p* < 0.001 (***), and *p* < 0.0001 (****); ns indicates not significant.

Compared to PBS, both vvDD and vvDD-nsmDR-18 effectively inhibited tumor growth by Day 34 after tumor inoculation (Figure 3A,B). Treatment with vvDD significantly prolonged survival of tumor-bearing mice compared to the PBS control (Figure 3C). Notably, vvDD-nsmDR-18 conferred superior antitumor efficacy, resulting in a significantly greater survival benefit relative to vvDD (Figure 3C). Median survival was 38 days for PBS-treated mice and 86 days for vvDD-treated mice, whereas median survival was not reached in the vvDD-nsmDR-18 group during the 110-day observation period (Figure 3C). Furthermore, necropsy on day 110 revealed no observable tumor nodules in the surviving mice that received oVV treatment.

Because the ID8a-luc model is characterized by extensive ascites formation [55], abdominal girth was measured as a surrogate of disease burden. On day 29 post tumor inoculation (day 24 posttreatment), mice receiving viral therapy exhibited significantly reduced abdominal distension compared with PBS-treated controls (Figure 3D). In a follow-up cohort, 4 of 8 vvDD-treated mice developed marked abdominal enlargement (>33 mm) (Figure 3E), whereas only 2 of 10 vvDD-nsmDR-18-treated mice exceeded this threshold (Figure 3F), compared with a normal girth of ~20 mm in healthy mice.

Collectively, these findings demonstrate that vvDD-nsmDR-18 treatment markedly enhances survival and was associated with reduced abdominal distension consistent with decreased ascites burden, a major clinical challenge in advanced ovarian cancer.

### 3.4. vvDD-nsmDR-18 Activates T Cells in the Spleen

**Figure 4:**
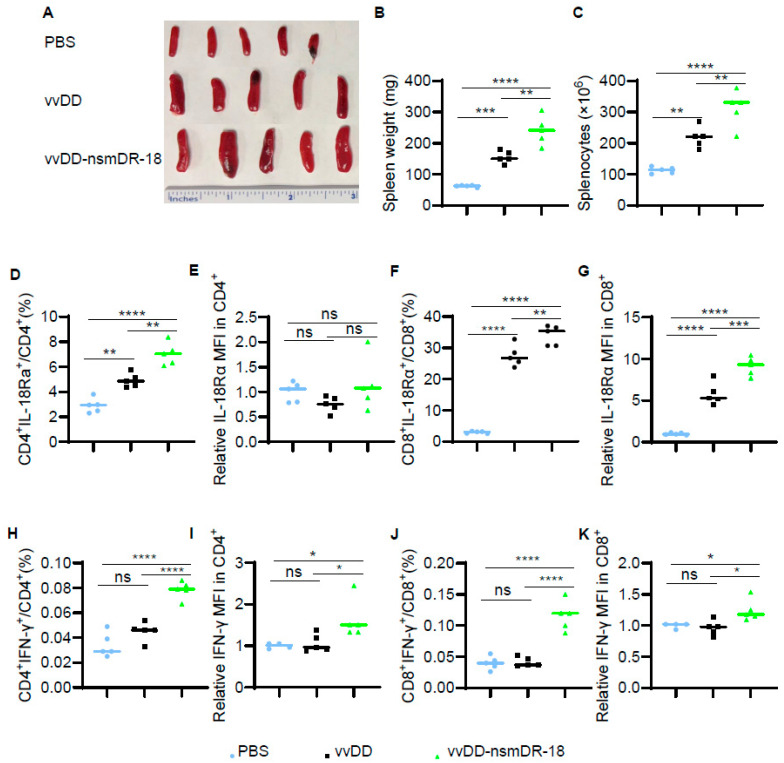
vvDD-nsmDR-18 activates T cells in the spleen. B6 mice were inoculated i.p. with 3.5 × 10^6^ ID8a-luc cells and treated i.p. with the indicated viruses (1.0 × 10^8^ PFU/mouse) or PBS five days after tumor cell inoculation (5 mice/group). Spleens were harvested, photographed (**A**), and weighed (**B**). Total splenocyte numbers are shown in (**C**). Single splenocytes were analyzed by flow cytometry, and the frequency and MFI of IL-18Rα and IFN-γ on T cells are shown in (**D**–**K**). Data show one representative experiment. *p* < 0.05 (*); *p* < 0.01 (**); *p* < 0.001 (***), and *p* < 0.0001 (****); ns indicates not significant.

To elucidate the mechanism underlying vvDD-nsmDR-18-induced antitumor activity in the ID8a-luc model, we first examined T cell responses in the spleen. Spleens from virus-treated mice were significantly enlarged compared with those from PBS-treated controls, and nsmDR-18 expression further increased spleen weight relative to vvDD alone (Figure 4A,B). Consistent with this observation, total splenocyte numbers increased proportionally with spleen weight (Figure 4C).

Single-cell suspensions from spleens were analyzed by flow cytometry. We first assessed IL-18Rα expression on T cells. Compared with PBS treatment, vvDD significantly increased the frequency of IL-18Rα^+^CD4^+^ T cells, while vvDD-nsmDR-18 induced a more pronounced increase (Figure 4D). However, the median fluorescence intensity (MFI) of IL-18Rα on CD4^+^ T cells was not significantly altered following viral treatment (Figure 4E). In contrast, both the frequency and MFI of IL-18Rα expression on splenic CD8^+^ T cells were significantly upregulated after viral treatment, with vvDD-nsmDR-18 producing a markedly stronger effect than vvDD alone (Figure 4F,G). These data indicate that both vaccinia virus infection and nsmDR-18 expression enhance IL-18 receptor availability on splenic T cells, particularly within the CD8^+^ compartment.

We next assessed splenic T cell activation by measuring IFN-γ production. Compared with PBS, vvDD alone did not significantly increase either the frequency or MFI of IFN-γ expression in splenic CD4^+^ or CD8^+^ T cells. In contrast, vvDD-nsmDR-18 markedly increased both the percentage and MFI of IFN-γ-producing CD4^+^ and CD8^+^ T cells (Figure 4H–K). These findings indicate that expression of nsmDR-18 is associated with enhanced splenic T cell activation following viral treatment.

### 3.5. vvDD-nsmDR-18 Activates T Cells in Peritoneal Lavage-Derived Cells

**Figure 5:**
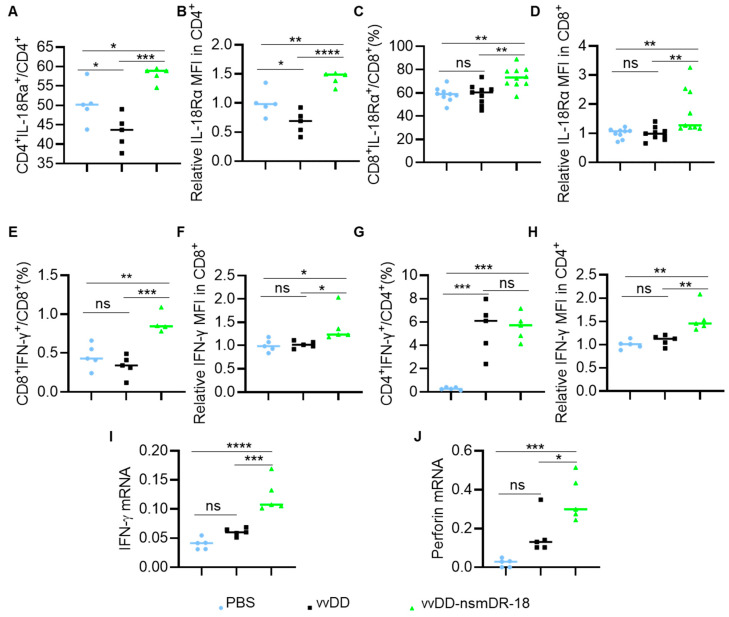
vvDD-nsmDR-18 activates T cells in the peritoneal lavage-derived cells. B6 mice were inoculated i.p. with 3.5 × 10^6^ ID8a-luc cells and treated i.p. with the indicated viruses (1.0 × 10^8^ PFU/mouse) or PBS five days after tumor cell inoculation (4–5 mice/group). Peritoneal lavage-derived cells were harvested and analyzed by flow cytometry to determine the frequency and MFI of IL-18Rα and IFN-γ expression on T cells (**A**–**H**). Peritoneal lavage-derived cells were also analyzed by RT-qPCR, and the expression of the effector molecules IFN-γ and perforin is shown in (**I**,**J**). Data show one representative experiment, except for panels (**C**,**D**), which present combined data from two independent experiments. *p* < 0.05 (*); *p* < 0.01 (**); *p* < 0.001 (***), and *p* < 0.0001 (****); ns indicates not significant.

We next evaluated T cell responses in peritoneal lavage-derived cells. The cells were collected by peritoneal lavage and analyzed by flow cytometry and RT-qPCR. Flow cytometric analysis revealed that vvDD-nsmDR-18, but not vvDD, significantly increased both the frequency and MFI of IL-18Rα expression on CD4^+^ and CD8^+^ T cells compared with PBS treatment (Figure 5A–D). Moreover, vvDD-nsmDR-18 significantly increased both the percentage and MFI of IFN-γ-producing CD8^+^ T cells (Figure 5E,F).

Interestingly, both viruses induced comparable increases in the frequency of IFN-γ^+^CD4^+^ T cells relative to PBS; however, vvDD-nsmDR-18 uniquely enhanced IFN-γ expression intensity (MFI) within this population (Figure 5G,H). Consistent with these findings, RT-qPCR analysis of peritoneal lavage-derived cells demonstrated that vvDD-nsmDR-18, but not vvDD, significantly elevated transcript levels of key effector molecules, including IFN-γ and perforin (Figure 5I,J). The enrichment of IFN-γ-producing CD8^+^ T cells and increased expression of cytotoxic effector genes likely contribute to the improved control of abdominal distension observed following vvDD-nsmDR-18 treatment.

### 3.6. vvDD-nsmDR-18 Activates T Cells Within Tumor Microenvironment

**Figure 6:**
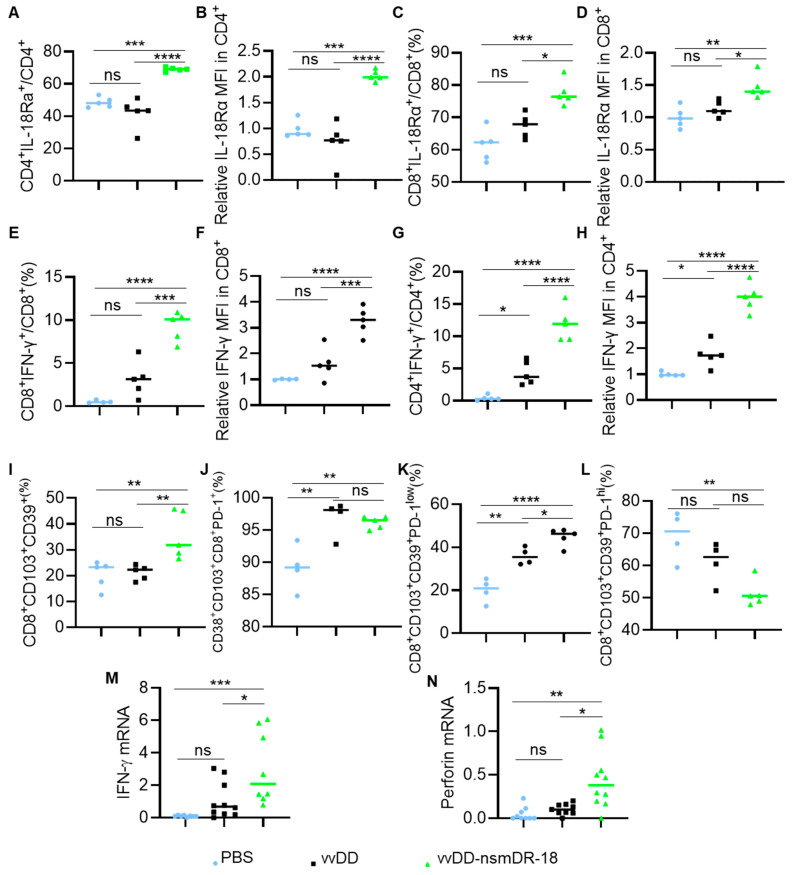
vvDD-nsmDR-18 activates T cells in the tumor. B6 mice were inoculated i.p. with 3.5 × 10^6^ ID8a-luc cells and treated i.p. with the indicated viruses (1.0 × 10^8^ PFU/mouse) or PBS five days after tumor cell inoculation (*n* = 4–6). Tumors were harvested to prepare single-cell suspensions for flow cytometry or RT-qPCR analysis. The frequency and MFI of IL-18Rα and IFN-γ on tumor-infiltrating T cells are shown in (**A**–**H**). The frequency of CD39^+^CD103^+^CD8^+^ T cells is shown in (**I**) and PD-1 expression on CD39^+^CD103^+^CD8^+^ T cells is shown in (**J**–**L**). Expression of effector molecules IFN-γ and perforin is shown in (**M**,**N**). Data show one representative experiment, except for panels (**M**,**N**), which present combined data from two independent experiments. *p* < 0.05 (*); *p* < 0.01 (**); *p* < 0.001 (***), and *p* < 0.0001 (****); ns indicates not significant.

We further characterized T cell activation within the tumor microenvironment. Single-cell suspensions prepared from tumor nodules were analyzed by flow cytometry. The patterns of IL-18Rα expression on tumor-infiltrating T cells and IFN-γ expression on CD8^+^ T cells closely mirrored those observed in peritoneal lavage-derived cells (Figure 6A–F).

Notably, both vvDD and vvDD-nsmDR-18 increased the frequency and MFI of IFN-γ expression in tumor-infiltrating CD4^+^ T cells relative to PBS treatment; however, vvDD-nsmDR-18 induced significantly stronger IFN-γ production than vvDD alone (Figure 6G,H). In addition, vvDD-nsmDR-18 significantly increased the abundance of highly activated, tumor-reactive CD8^+^ T cells (CD39^+^CD103^+^CD8^+^) (Figure 6I), consistent with our previous observations in a murine colon cancer model [52].

The CD39^+^CD103^+^CD8^+^ T cells exhibited high PD-1 expression, consistent with an “exhausted” yet functionally potent phenotype. While total PD-1 expression on CD39^+^CD103^+^CD8^+^ T cells did not differ between vvDD and vvDD-nsmDR-18 treatments (Figure 6J), vvDD-nsmDR-18 significantly increased the proportion of CD39^+^CD103^+^CD8^+^PD-1^low^ T cells compared with other treatments and reduced the proportion of CD39^+^CD103^+^CD8^+^PD-1^high^ T cells compared with PBS treatment (Figure 6K,L). Although the reduction in CD39^+^CD103^+^CD8^+^PD-1^high^ T cells did not reach statistical significance relative to vvDD, this shift may suggest improved functional fitness of tumor-reactive CD39^+^CD103^+^CD8^+^ T cells following nsmDR-18 delivery.

Finally, RT-qPCR analysis of tumor tissues confirmed that vvDD-nsmDR-18 significantly increased the expression of key effector molecules, including IFN-γ and perforin, compared with control treatments (Figure 6M,N), further supporting its role in enhancing intratumoral T cell cytotoxicity.

### 3.7. Safety Observations and Splenomegaly

No overt signs of systemic toxicity were observed in virus-treated animals based on routine clinical monitoring, including activity, posture, grooming behavior, and body condition. Body weight was not used as a toxicity indicator due to the confounding effects of ascites accumulation in this model. At necropsy, no obvious gross pathological abnormalities were detected in major organs, including liver, lungs, kidneys, and heart, in virus-treated animals compared with controls. Consistent with immune activation, splenomegaly and increased splenocyte numbers were observed, particularly in the vvDD-nsmDR-18-treated group.

## 4. Discussion

Ovarian cancer is the most lethal gynecologic malignancy, largely due to late diagnosis, as early symptoms are often nonspecific or absent until the disease has progressed to advanced stages. More than 90% of patients develop malignant ascites, a hallmark of advanced ovarian cancer that is notoriously difficult to control and contributes substantially to morbidity and treatment failure. Despite improvements in surgery and chemotherapy, durable disease control in advanced ovarian cancer remains difficult to achieve, highlighting the need for innovative therapeutic strategies capable of overcoming the highly immunosuppressive peritoneal tumor microenvironment.

Cytokines are central regulators of antitumor immunity and have emerged as powerful modulators in cancer immunotherapy, making them compelling candidates for therapeutic intervention. IL-18, a member of IL-1 family, is synthesized as an inactive precursor and requires intracellular processing by caspase-1 within the NLRP3 inflammasome to generate its biologically active form [56]. To overcome endogenous inhibition by IL-18 binding protein and reduce systemic toxicity, we generated the vvDD-nsmDR-18 to deliver nsmDR-18 while maximizing localized immune activation with reduced off-target effects [52]. In the present study, we investigated the therapeutic potential vvDD-nsmDR-18 in an aggressive intraperitoneal ovarian cancer model. Treatment with vvDD-nsmDR-18 was associated with prolonged survival and reduced abdominal distension consistent with decreased ascites burden.

To elucidate the mechanisms underlying vvDD-nsmDR-18-induced antitumor activity, we evaluated T cell activation across multiple anatomical compartments. Our data show that nsmDR-18 delivered via oVV significantly upregulates IL-18Rα not only on tumor-infiltrating T cells (Figure 6A–D), consistent with prior reports [52,57], but also on T cells in the spleen (Figure 4D,F,G) and peritoneal lavage-derived cells (Figure 5A–D). These findings suggest that nsmDR-18 may induce a positive feedback loop that systemically enhances IL-18 responsiveness across distinct immune niches. Correspondingly, elevated IL-18Rα expression was associated with increased IFN-γ production in these T cell populations (Figure 4H–K, Figure 5E–H and Figure 6E–H), indicating robust T cell activation and heightened antitumor effector potential. Mechanistically, delivery of nsmDR-18 through the oncolytic virus promoted immune activation across multiple anatomical compartments, including tumors, peritoneal lavage-derived cells, and spleens, accompanied by increased expression of effector molecules such as IFN-γ and perforin. These findings suggest that localized IL-18 signaling mediated by the engineered virus may enhance antitumor immune responses within the peritoneal tumor environment.

Interestingly, vvDD, especially vvDD-nsmDR-18, induced marked splenomegaly accompanied by expansion and activation of splenic T cells. This observation is mechanistically intriguing in light of recent evidence identifying the spleen as a critical, and potentially primary, reservoir for tumor-infiltrating T cells, especially following immune checkpoint blockade [58]. Our findings support the concept that vvDD-nsmDR-18 may mobilize splenic T cell pools to sustain antitumor immunity, thereby contributing to durable tumor control. Although our findings are consistent with this emerging concept, the present data remain correlative and do not establish the spleen as a direct source of tumor-infiltrating T cells in this model.

Consistent with our previous studies, vvDD-nsmDR-18 treatment significantly increased the frequency of tumor-reactive CD39^+^CD103^+^CD8^+^ T cells in the ID8a-luc ovarian cancer model. Co-expression of CD39 and CD103 marks a subset of tumor-infiltrating CD8^+^ T cells with enhanced cytolytic capacity and strong associations with improved clinical outcomes [59,60,61,62]. Although CD39^+^CD103^+^CD8^+^ T cells typically exhibit high PD-1 expression [63], our data reveal that while both vvDD and vvDD-nsmDR-18 increase PD-1^+^ frequencies in CD39^+^CD103^+^CD8^+^ T cells, nsmDR-18 skews this population toward a PD-1^low^ phenotype. However, these observations represent associations rather than direct causal evidence for their role in mediating therapeutic benefit. Moreover, PD-1 expression was assessed at a single time point, and longitudinal analyses will be required to determine whether this phenotype reflects sustained T cell functionality or delayed exhaustion.

It is important to acknowledge several limitations of the present study. First, the ID8 intraperitoneal ovarian cancer model recapitulates key features of advanced human disease, including peritoneal dissemination and ascites formation, but cannot fully capture the genetic heterogeneity and clinical variability of human ovarian cancer. Therefore, evaluation of vvDD-nsmDR-18 in additional ovarian cancer models with diverse molecular backgrounds will be important to determine the broader applicability of this approach.

Second, abdominal distension (body girth) was used as a surrogate indicator of ascites burden. Although this measurement is commonly used in intraperitoneal ovarian cancer models, it can be influenced by factors such as tumor burden and physiological variability. To aid interpretation, qualitative necropsy observations were recorded and generally showed reduced abdominal distension and less visible peritoneal fluid accumulation in virus-treated animals compared with controls. Nonetheless, direct quantification of ascites volume would further strengthen these findings and will be incorporated in future studies.

Third, although vvDD-nsmDR-18 treatment was associated with increased IL-18Rα expression and elevated levels of IFN-γ and perforin, the current data remain correlative and do not establish the essential immune mediators responsible for therapeutic efficacy. In addition to CD8^+^ T cells, IL-18 is known to activate several immune populations, including NK cells, whose potential contribution was not directly evaluated in this study. Future mechanistic experiments will be required to determine the relative roles of these immune cell populations.

Finally, splenomegaly observed following vvDD-nsmDR-18 treatment is consistent with systemic immune activation; however, alternative explanations, including inflammatory responses, cannot be excluded. Importantly, no overt clinical signs of toxicity were observed, and no gross pathological abnormalities were detected in major organs at necropsy. In this model, body weight is not a reliable indicator of systemic toxicity due to the confounding effects of ascites accumulation, which can lead to weight gain independent of health status. Therefore, clinical observations and gross pathology were prioritized for safety assessment. Nevertheless, these evaluations remain limited, and comprehensive safety studies, including histopathological and cytokine analyses, will be required in future work.

Given the immune-modulating properties of oncolytic viruses, an important future direction will be to evaluate vvDD-nsmDR-18 in combination with existing therapeutic modalities, including platinum-based chemotherapy and immune checkpoint inhibitors. Such strategies may further enhance antitumor immunity and improve clinical outcomes in ovarian cancer.

## 5. Conclusions

In conclusion, this study demonstrates that an oncolytic vaccinia virus expressing a non-secreted decoy-resistant IL-18 mutein can promote immune activation across multiple anatomical compartments and is associated with prolonged survival in an aggressive intraperitoneal ovarian cancer model. The treatment was also associated with reduced abdominal distension consistent with decreased ascites burden and expansion of tumor-reactive CD8^+^ T cell populations. Although additional mechanistic and safety studies are required, these findings support further investigation of vvDD-nsmDR-18 as a potential immunotherapeutic strategy for ovarian cancer.

## Data Availability

All data is available from the corresponding author upon reasonable request.
